# Supplementary material for: Revealing the room temperature superplasticity in bulk recrystallized molybdenum
Source: Nat Commun. 2023 Dec 14;14:8336. doi: 10.1038/s41467-023-44056-7 (PMC10721596; doi:10.1038/s41467-023-44056-7)
Supplement: Supplementary file 1 — Supplementary Information [file 41467_2023_44056_MOESM1_ESM.docx]

**Supplementary Information**

**Revealing the Room Temperature Superplasticity in Bulk Recrystallized Molybdenum**

Wenshuai Chen^1,2,3,#^, Xiyao Li^4,#^, Shenbao Jin^5^, Lunwei Yang^2^, Yan Li^2^，Xueliang He^2^, Wanting Zhang^1,2,3^, Yinxing Wu^5^, Zhilin Hui^2^, Zhimin Yang^1,2,3^, Jian Yang^2^, Wei Xiao^2,3,6^, Gang Sha^5,^*, Jiangwei Wang^4,^*, Zenglin Zhou^1,2,3,^*

**This supplementary information includes:**

Supplementary Table 1

Supplementary Figures 1-17

Supplementary Discussion

Supplementary References

**Supplementary Table**

| **Bond** | | **Bond order** | | |
| --- | --- | --- | --- | --- |
|  |  | **GB+1O_int_** | **GB+4Ni_sub_** | **GB+4Ni_sub_+1O_int_** |
| **Mo-Mo** | **1-2** | 0.337 | 0.392 | 0.333 |
|  | **1-3** | 0.317 | 0.392 | 0.198 |
|  | **1-4** | 0.173 | 0.388 | 0.403 |
|  | **1-5** | 0.191 | 0.225 | 0.190 |
|  | **1-6** | 0.323 | 0.350 | 0.350 |
|  | **1-7** | 0.312 | 0.350 | 0.270 |
|  | **1-8** | 0.203 | 0.365 | 0.342 |
|  | **1-9** | 0.189 | 0.202 | 0.208 |
| **Mo-Ni** | **1-10** | — | 0.471 | 0.213 |
|  | **1-11** | — | 0.471 | 0.499 |
|  | **1-12** | — | 0.471 | 0.438 |
| **Mo-O** | **1-13** | 0.498 | — | 0.432 |

**Supplementary Table 1 The bond order for GB cohesion with the segregation of O and Ni.**

**Supplementary Figures**


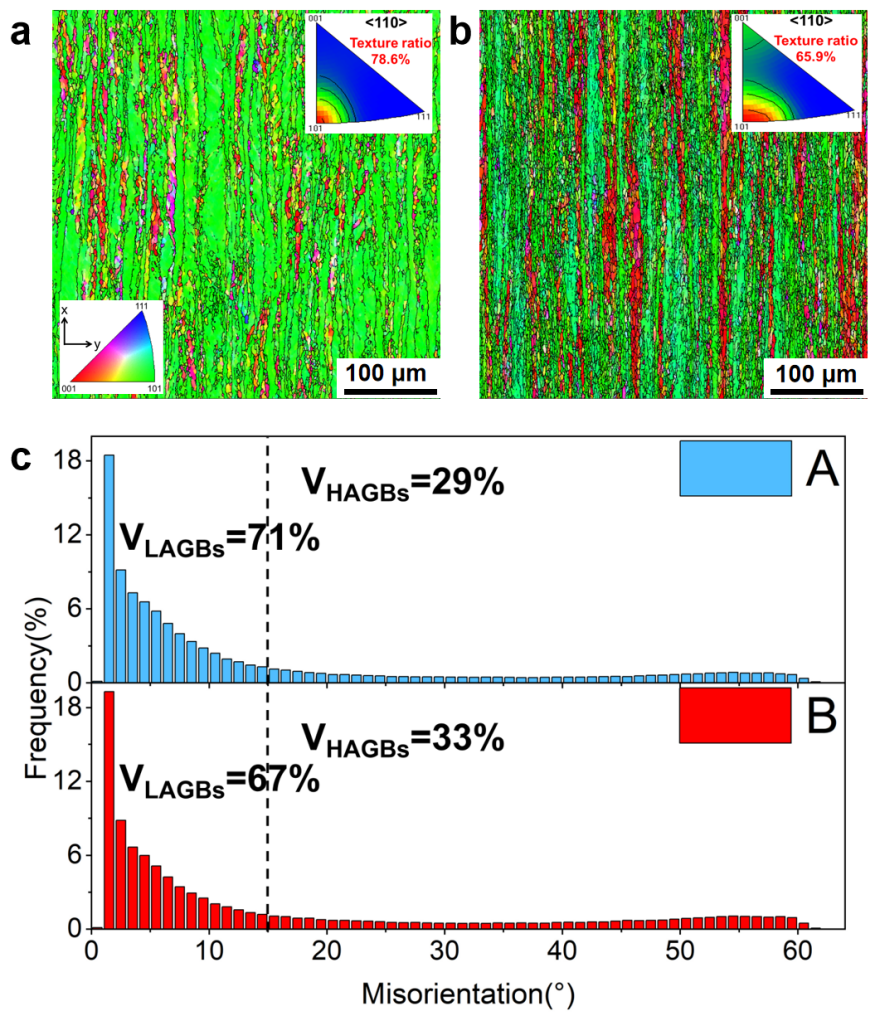


**Supplementary Fig. 1** **Microstructure and GB distribution of hot-rolled samples A and B. a, b** The microstructure and texture of **a** sample A and **b** sample B, respectively**. c** The GB distribution of samples A and B. These two Mo bars show a slender fibrous structure along the rolling direction. The sample B was deformed to a higher degree, and thus its fibrous grains are more elaborate and uniform. The ratio of <110>//RD main texture and LAGBs formed in sample B by thermal mechanical processing, is also slightly lower than that of sample A.


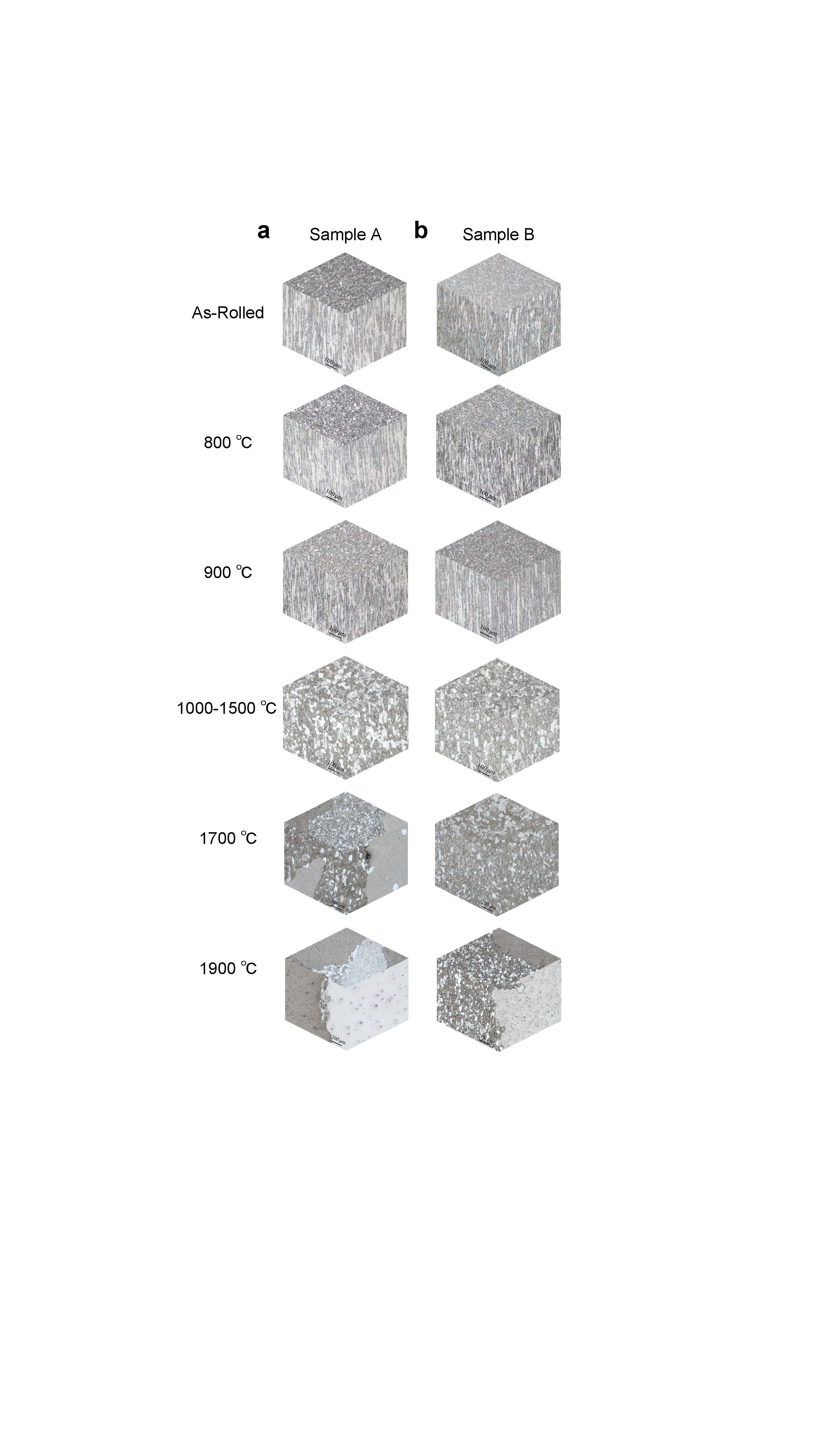


**Supplementary Fig. 2 Three-dimensional metallographic microstructures of samples A and B after annealing at different temperatures.** Apparent recrystallization occurred with the increase of annealing temperature. After annealing at 1000 ℃, the recrystallization process of both **a** sample A and **b** sample B is basically completed. Sample A showed abnormal grain growth once annealed above 1700 ℃, while sample B started to exhibit abnormal grain growth above 1900 ℃.


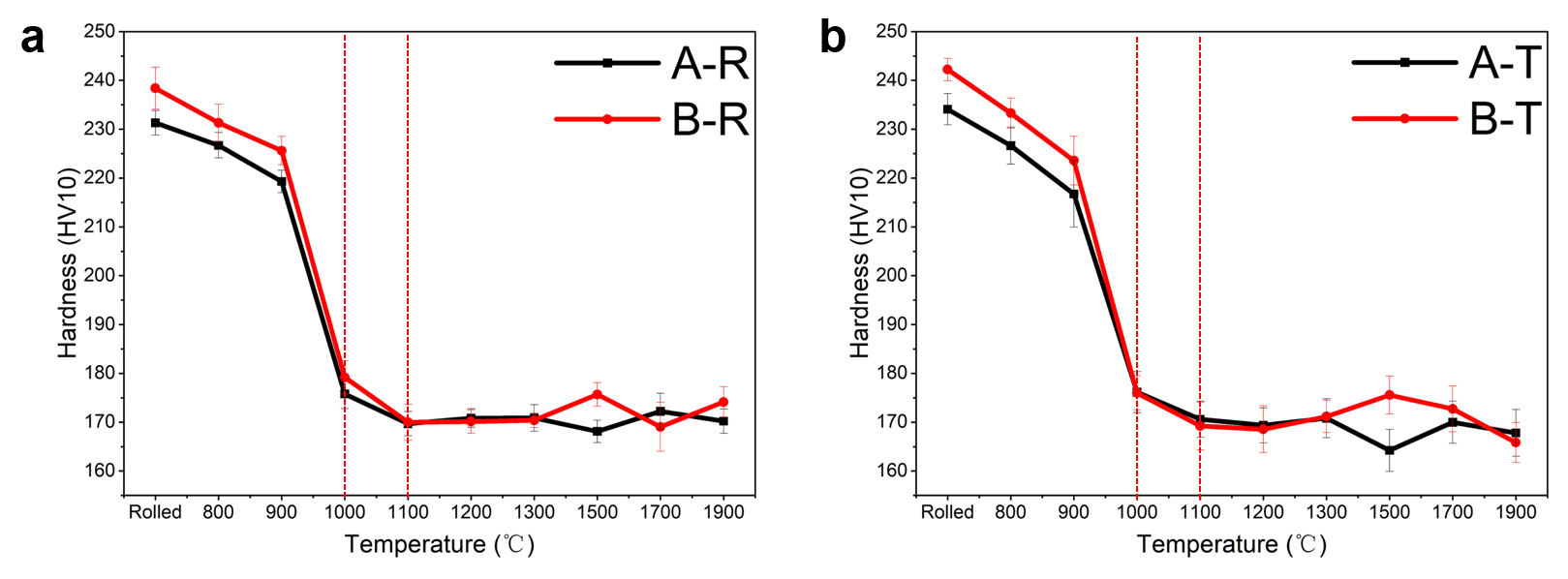


**Supplementary Fig. 3 Vicker hardness HV_10_ of as-rolled and annealed samples A and B at 800~1900 ℃.** **a** The Vicker hardness in the R direction of samples A and B **(**R represents the longitudinal section along the rolling direction). **b** The Vicker hardness in the T direction of samples A and B (T represents the cross section perpendicular to the rolling direction). Both samples A and B completed more than 90 % of the recrystallization process after annealing at 1000 ℃, and experienced the full recrystallization process in 1100 ℃ and higher temperature annealed states.


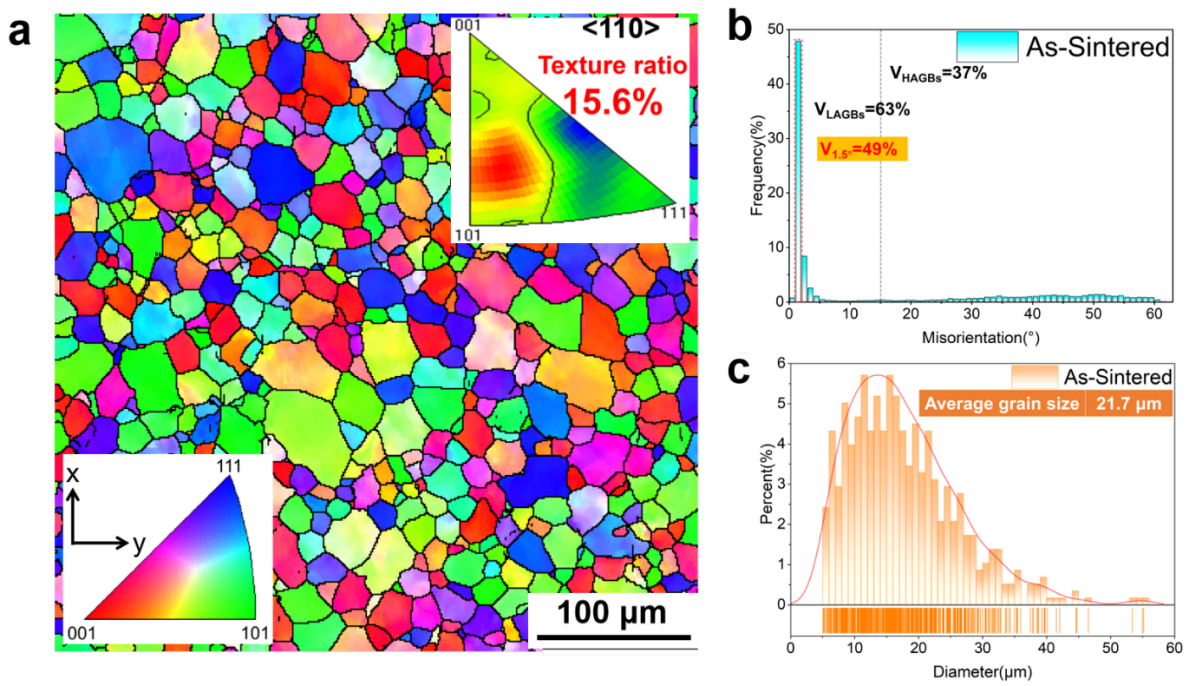


**Supplementary Fig. 4** **Microstructure, GB distribution and grain size distribution of sintered Mo bar. a** Microstructure and texture of sintered Mo bar. **b** GB distribution of sintered Mo bar. **c** Grain size distribution of sintered Mo bar. The sintered Mo presents equiaxed grains with random orientation distribution, and the ratio of <110>//RD texture is only 15.6 %. The average grain size is 21.7 μm. The proportion of LAGB reaches 63 %, of which 1.5° GBs account for 49 %.


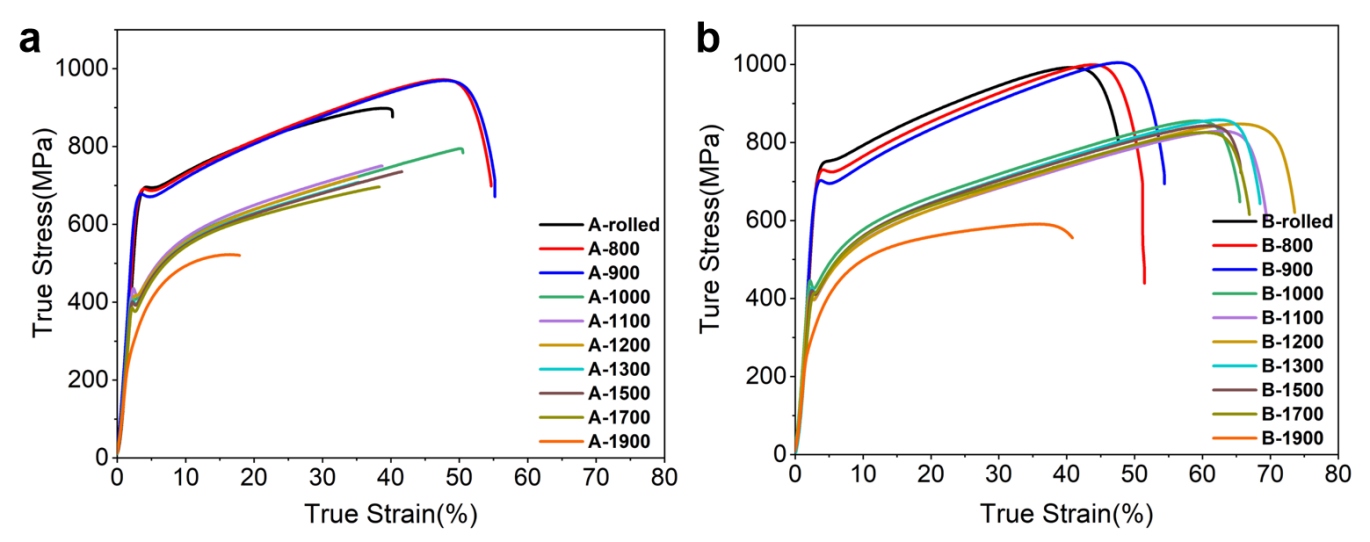


**Supplementary Fig. 5 True stress-strain curves of as-rolled and annealed samples A and B. a, b** True stress-strain curves of as-rolled and annealed **a** sample A and **b** sample B, respectively.


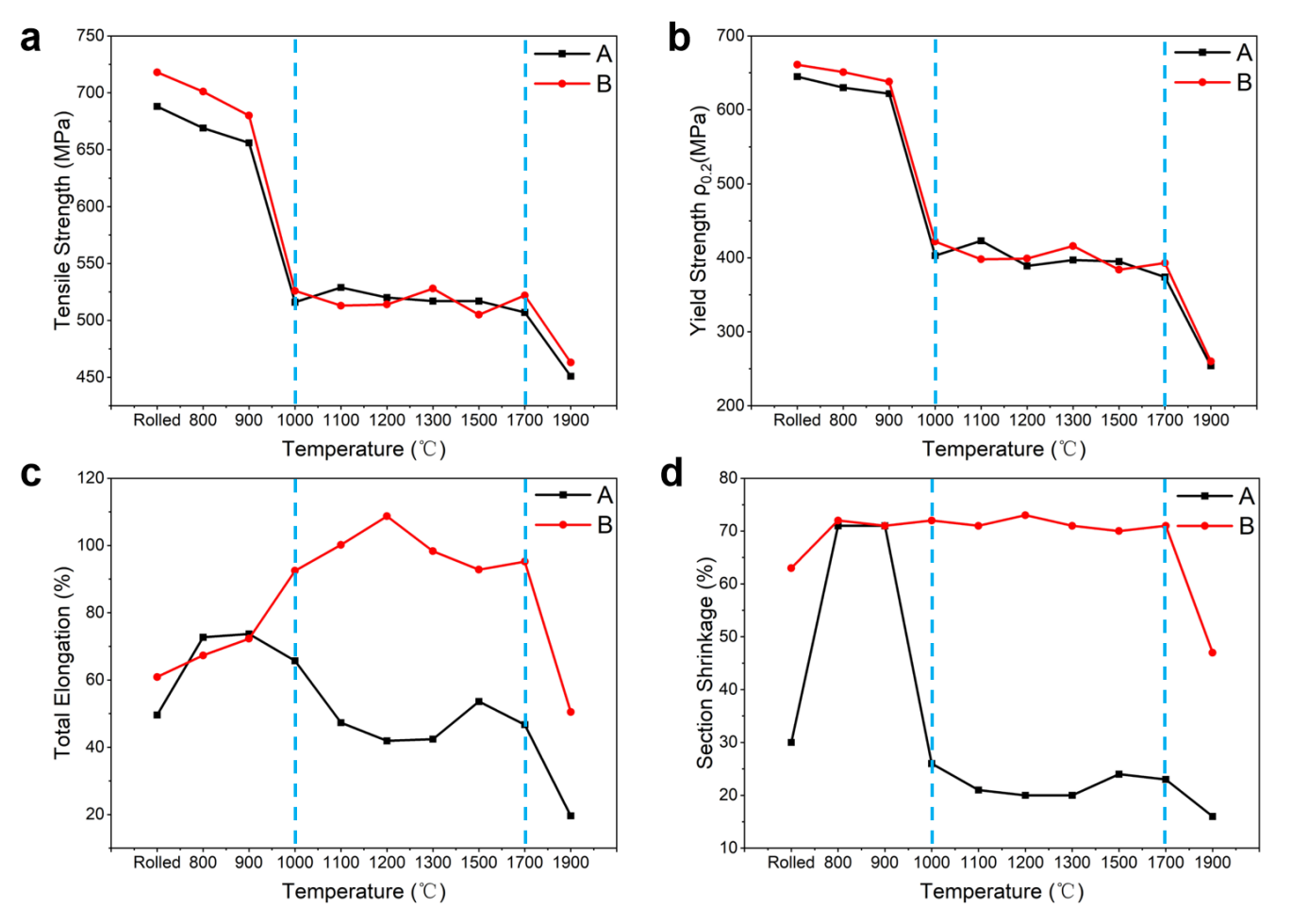


**Supplementary Fig. 6 Mechanical properties of samples A and B with different processing conditions. a** Tensile strength. **b** Yield strength. **c** Total elongation. **d** Reduction of area.


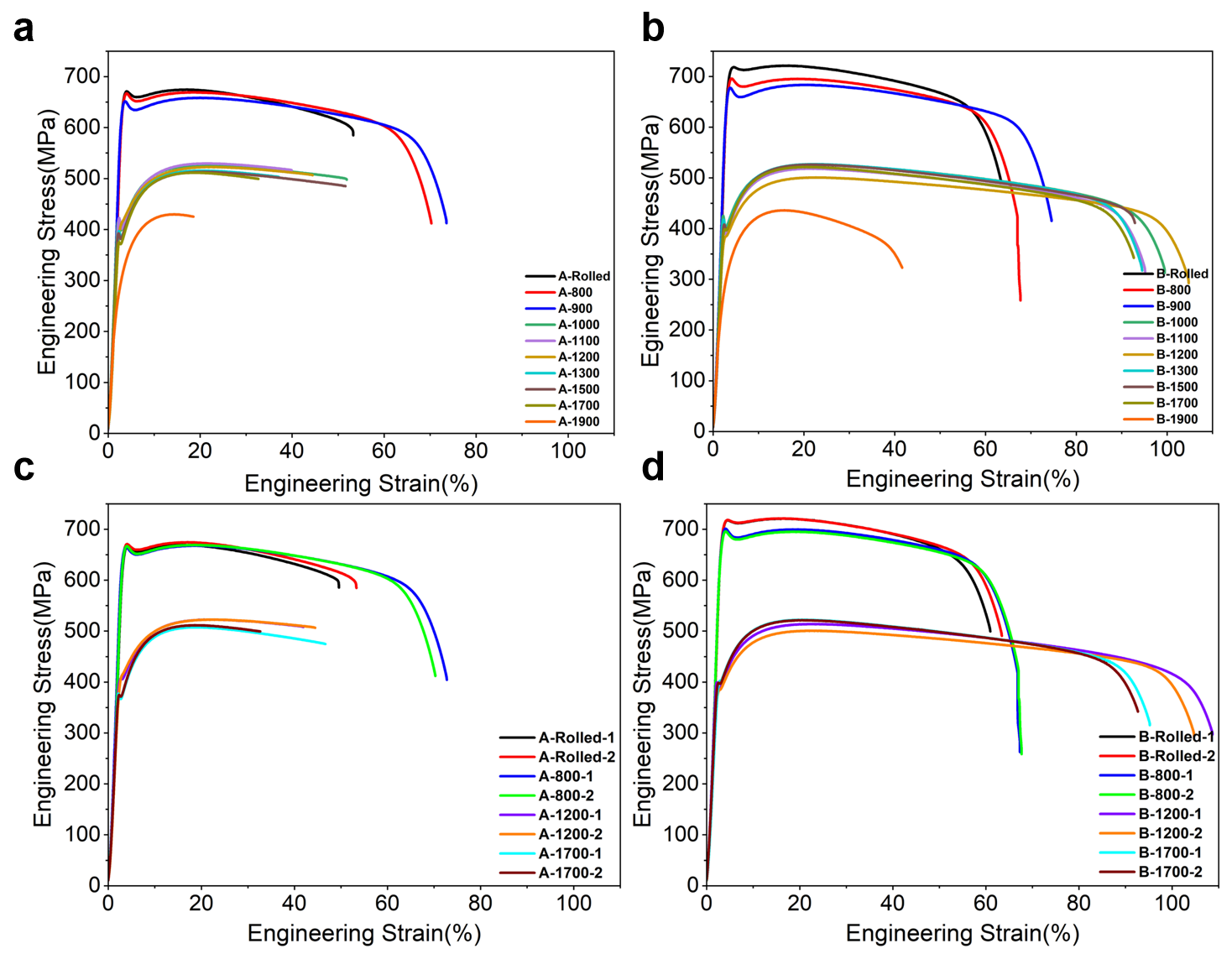


**Supplementary Fig. 7 Engineering stress-strain curves of different Mo samples. a, b** Engineering stress-strain curves of **a** sample A and **b** sample B after rolling and different annealing, respectively. **c-d** Parallel engineering stress-strain curves for samples A and B, respectively.


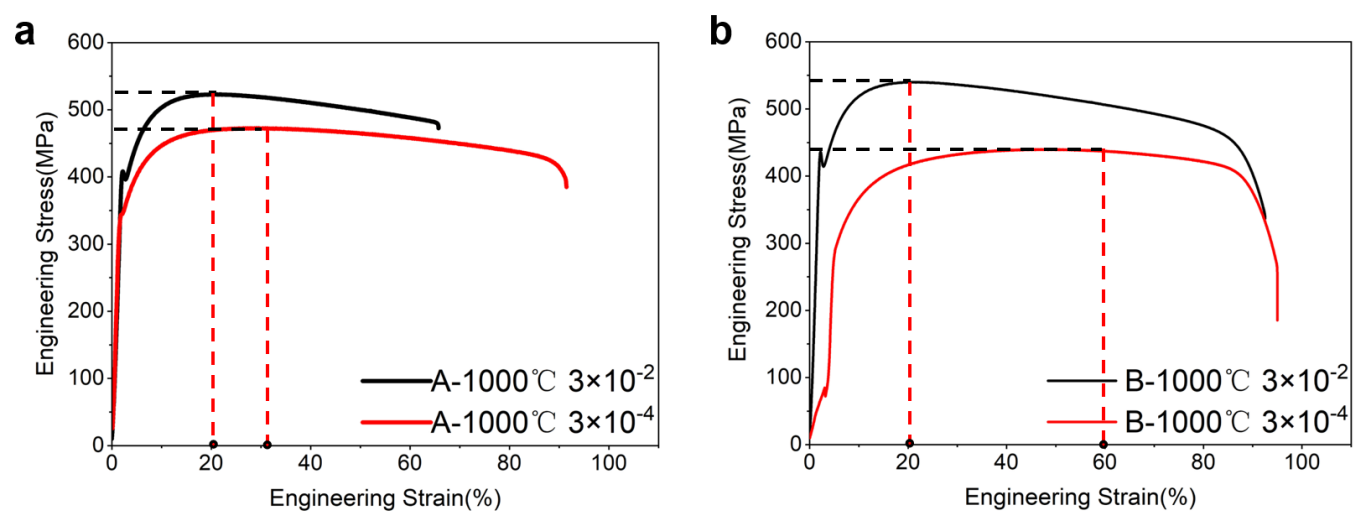


**Supplementary Fig. 8 Engineering stress-strain curves of samples A-1000 and B-1000 deformed under different tensile rates. a, b** Engineering stress-strain curves of **a** sample A-1000 and **b** sample B-1000 deformed under different tensile rates, respectively. When the constant strain rate decreases from 3×10^-2^ s^-1^ to 3×10^-4^ s^-1^, the uniform elongation of sample A-1000 increases from 20 % to 31 %, and the uniform elongation of sample B-1000 increases from 20 % to 60 %.


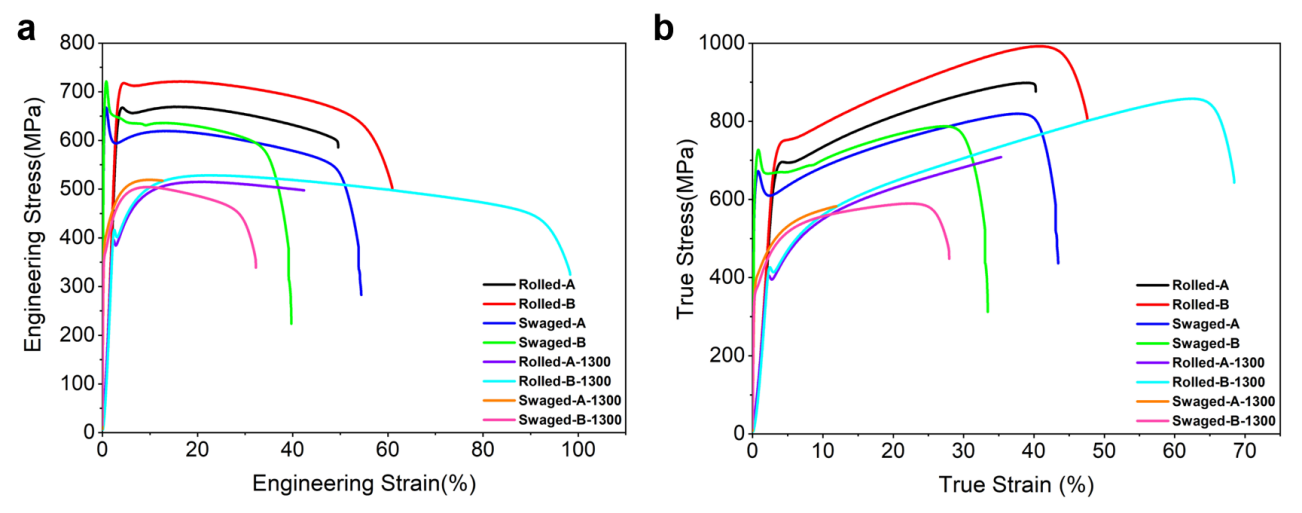


**Supplementary Fig. 9 Engineering stress-strain and true stress-strain curves of as-rolled and rotary-forged Mo bars under corresponding deformation and annealed at 1300 ℃. a** Engineering stress-strain curves**. b** True stress-strain curves. The strength of as-rolled Mo bars is significantly higher than that of rotary-forged Mo bars. The total elongation at room temperature of rolled-1300 ℃ annealed Mo bars is as high as 99 %, and that of rotary-forged and 1300 ℃ annealed Mo bars is only 30 %.


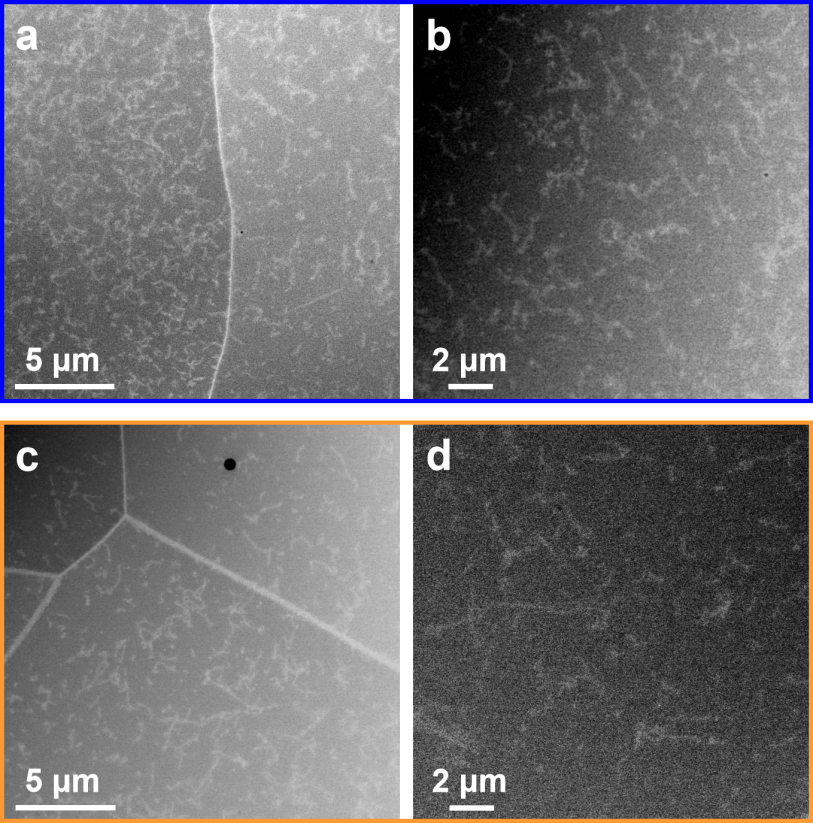


**Supplementary Fig. 10 Comparison of dislocation density in as-received sample A-1200 and sample B-1200. a, c** Dislocations distribution near grain boundaries in **a** sample A-1200 and **c** sample B-1200, respectively. **b, d** Intragranular dislocations distribution in **b** sample A-1200 and **d** sample B-1200, respectively. The intragranular dislocation configurations of samples A-1200 and B-1200 are similar, which are ρ_A-1200_=4×10^8^ /cm^2^ and ρ_B-1200_=7.9×10^7^ /cm^2^.


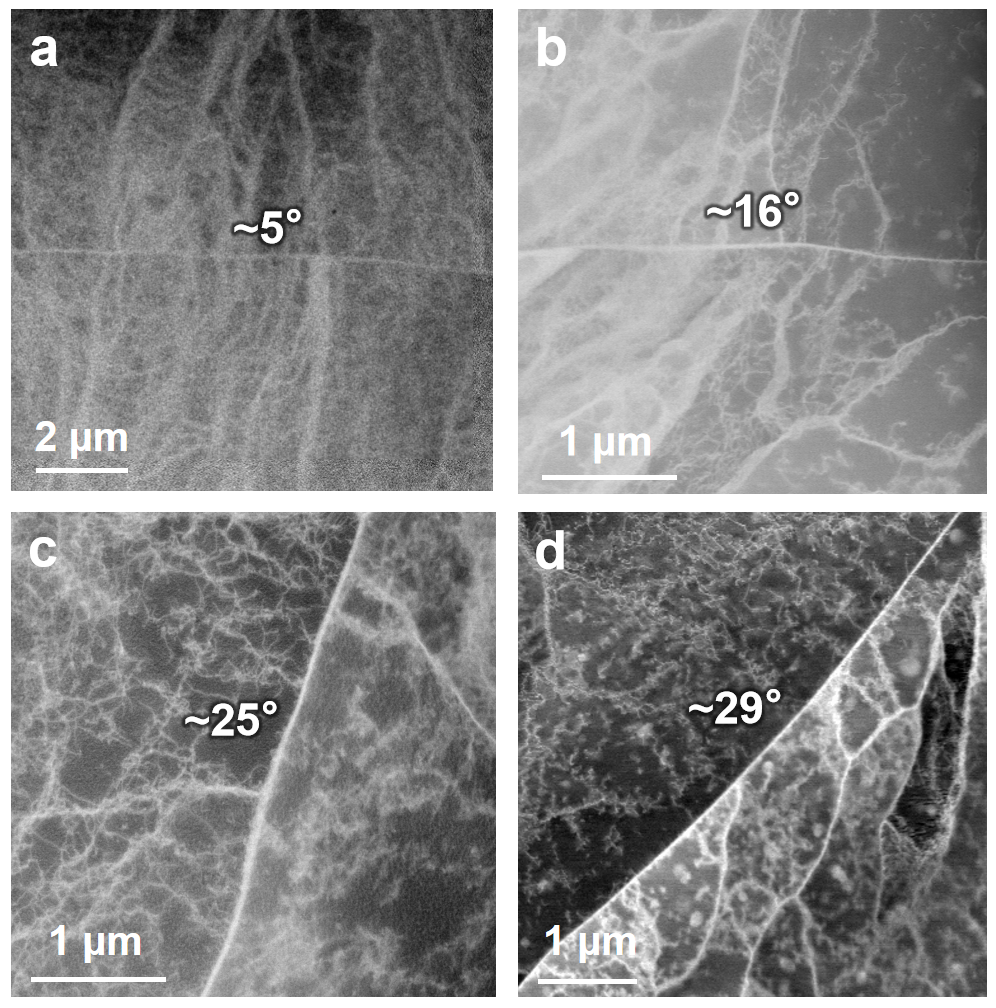


**Supplementary Fig. 11 Additional high-angle annular dark-field scanning transmission electron microscopy (HAADF-STEM) images showing the GB angle-dependent interaction of dislocation with GBs in sample B-1200.** The straight dislocation can move across **a** LAGBs but not for **b-d** HAGBs.

**
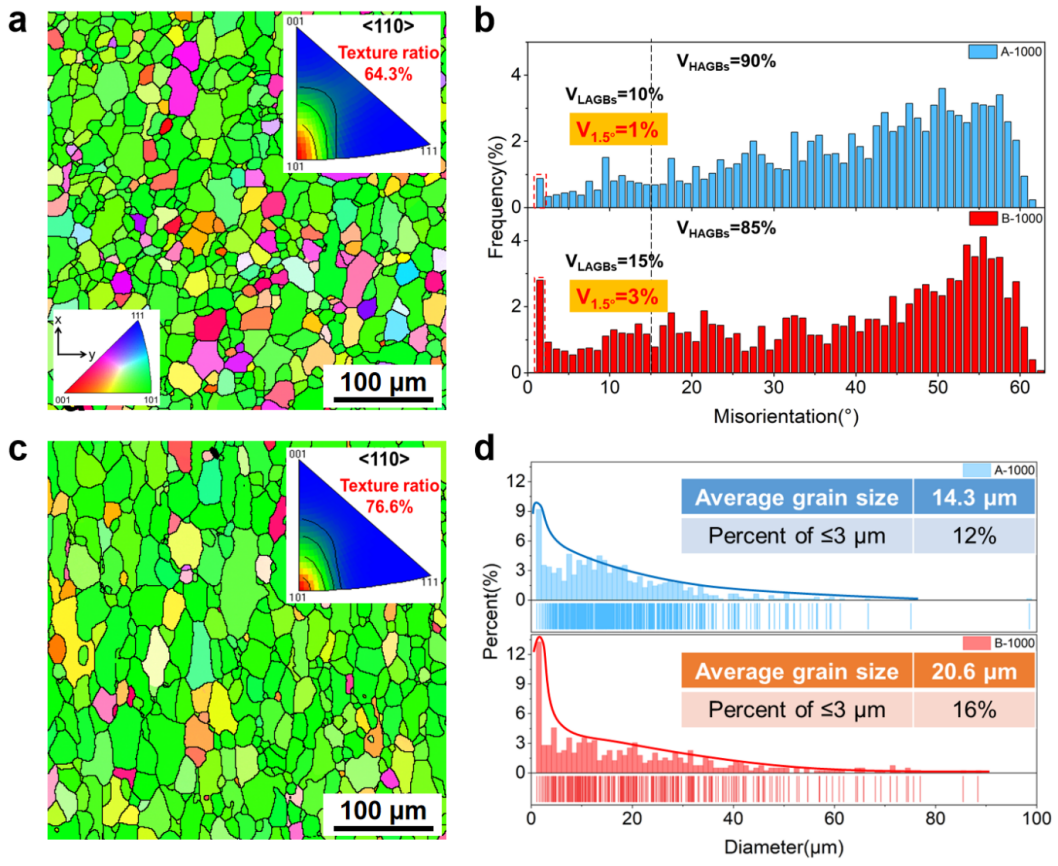
**

**Supplementary Fig. 12** **Microstructure, GB distribution and grain size distribution of A-1000 and B-1000 Mo bars. a, c** Microstructure and texture of **a** sample A-1000 and **c** sample B-1000, respectively. **b** GB distribution of samples A-1000 and B-1000. **d** Grain size distribution of samples A-1000 and B-1000.


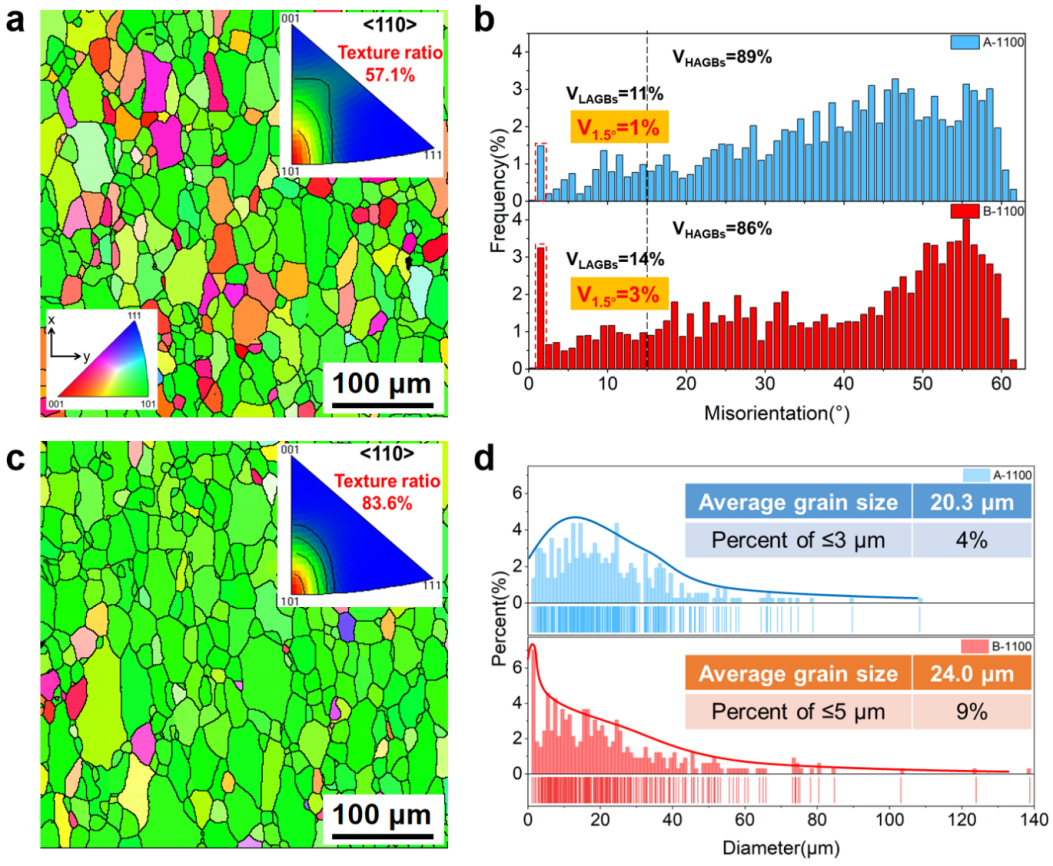


**Supplementary Fig. 13** **Microstructure, GB distribution and grain size distribution of A-1100 and B-1100 Mo bars. a, c** Microstructure and texture of **a** sample A-1100 and **c** sample B-1100, respectively. **b** GB distribution of samples A-1100 and B-1100. **d** Grain size distribution of samples A-1100 and B-1100.


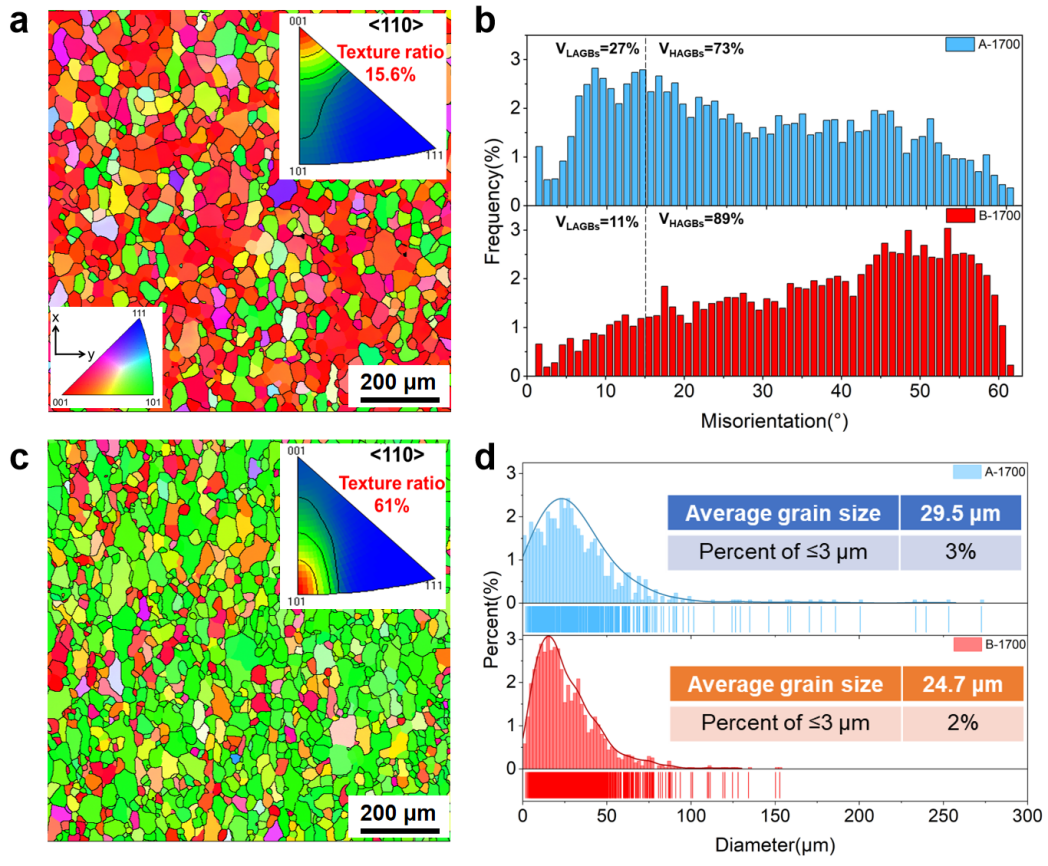


**Supplementary Fig. 14** **Microstructure, GB distribution and grain size distribution of A-1700 and B-1700 Mo bars. a, c** Microstructure and texture of **a** sample A-1700 and **c** sample B-1700, respectively. **b** GB distribution of samples A-1700 and B-1700. **d** Grain size distribution of samples A-1700 and B-1700.


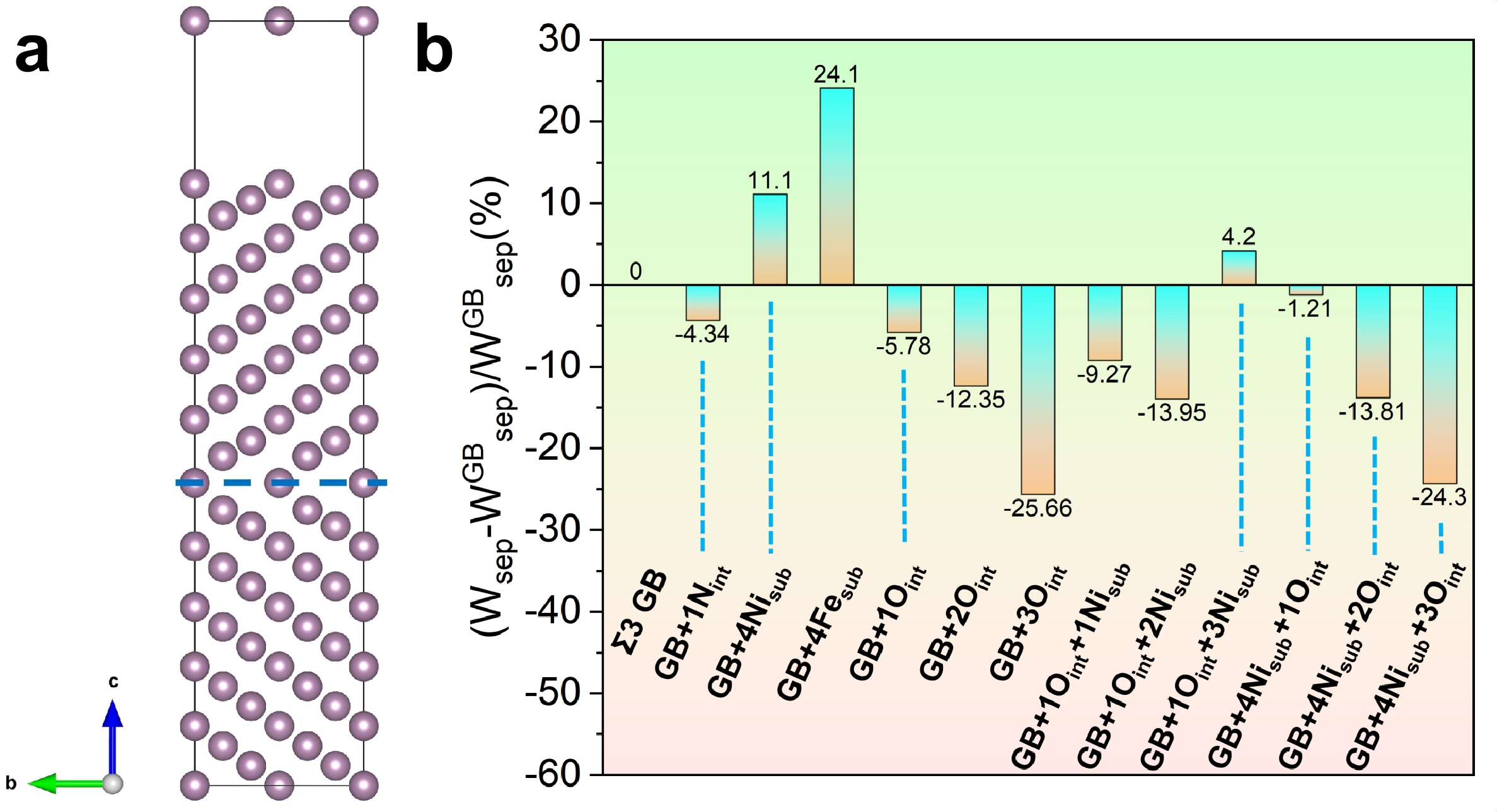


**Supplementary Fig. 15 Simulated structure of Mo Ʃ3(111)[110] GB and calculation of interface bonding of recrystallized Mo material. a** Simulated structure of a typical Mo Ʃ3(111)[110] GB containing interstitial atoms. **b** Relative separation work (percentage) of O, N, Ni, Fe element segregation and O, Ni co-segregation at the typical Mo Ʃ3(111)[110] GB, showing that the GB fracture strength decreases significantly with the increase of O concentration, while the co-segregation of Ni with O can help reducing the GB brittleness to some extent.


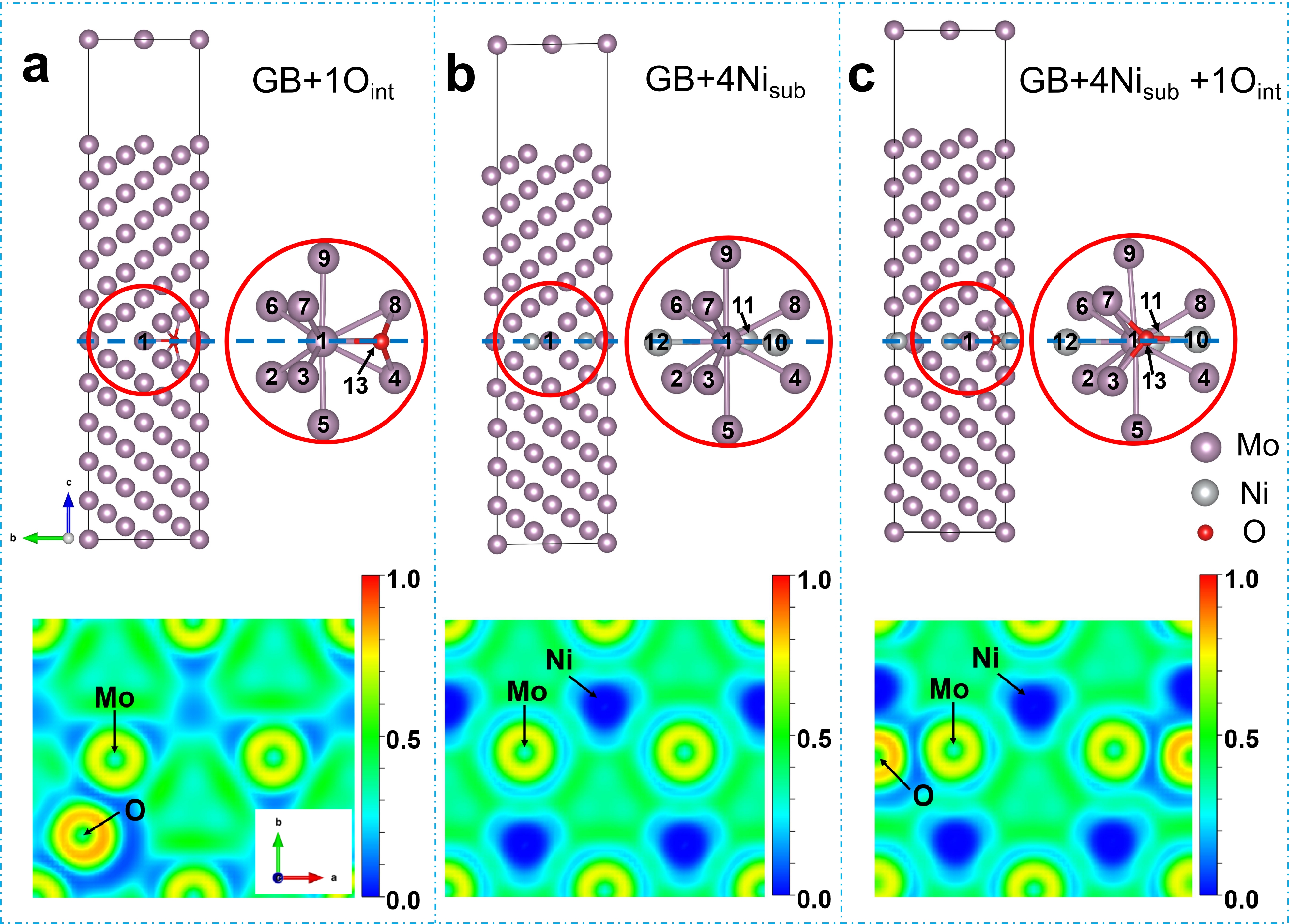


**Supplementary Fig. 16 Geometrical structures (top left), the enlarged core structures (top right) and the 2D ELF diagram (bottom) of Mo Ʃ3(111)[110] GB segregation with O and Ni atoms. a** Oxygen atom segregation at Mo GB. **b** the substituted Ni atoms. **c** O and Ni co-segregation at Mo GB. The red circle and Mo atom marked with 1 in geometrical structures indicates the magnified core structure at Mo GB center. The enlarged core structure of the GB center has been labeled with numbers around Mo atom, and their bond order values are listed in Supplementary Table 1. The 2D ELF diagram in **a** show that the color between interstitial O atom and adjacent metal atoms changes from orange (~0.8) to blue (~0.1), indicating that the outer electrons of metal atoms are captured by O and forming typical ionic bonds. The color around the substituted Ni atoms in **b** is close to wathet (0.3-0.4), forming new and stronger Mo-Ni metallic bonds, which is considered to enhance the GB adhesion. The 2D ELF diagram of **c** at Mo GB shows that the electron localization in the blue region between Mo-O is weakened, due to the additional free electrons brought by the segregated Ni atoms at the GBs. Therefore, the brittleness of the GBs caused by Mo-O bonding is decreased, which can help to increase its resistance against intergranular fracture.


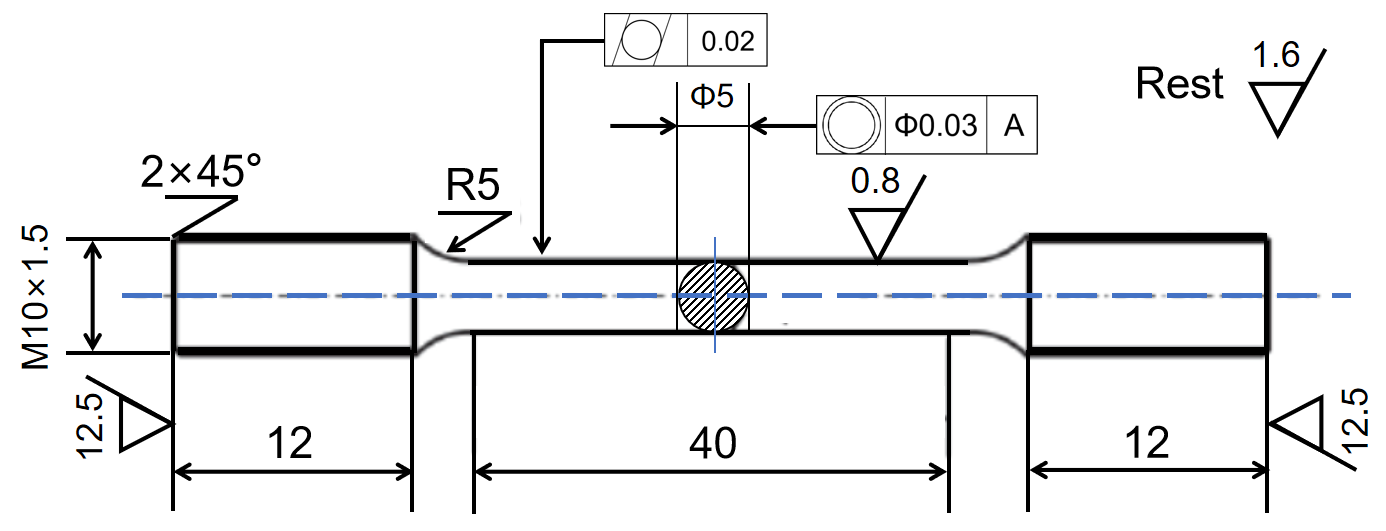


**Supplementary Fig. 17 Geometry of the dog-bone-shaped standard tensile specimen.** The parallel part length of the standard tensile specimen is 40 mm, with the diameter of 5 mm, and the original gauge distance is 25 mm.

**Supplementary Discussion:**

To reveal the effects of solute segregation on mechanical property of Mo, the first-principles calculations were conducted to study the cohesion strength of GBs, especially HAGBs that often dominate the intergranular fracture of Mo. A Mo Ʃ3(111)[110] GB with the segregations of substitutional Fe, Ni, interstitial O and N atoms was established using a coincidental site lattice (CSL) approach (Supplementary Fig. 15). The relaxed body-centered cubic (bcc) Mo unit cell has a cubic lattice parameter of 3.149 Å. The fully relaxed Mo Σ3 GB consists of 124 atoms, with the dimensions of 8.89 Å × 7.69 Å × 34.84 Å, and a vacuum thickness of 7.5 Å along the z-axis. Due to the higher stability of metal atoms near the central atom layers of the Mo GB, and the greater stability of oxygen atoms in the central layer^1-3^, we calculated the possible element segregation positions of O, Ni and Fe atoms in the central layer and its surrounding layers of the Mo GB, with a total of 5 layers. We found that when interstitial O atom and substitutional Ni atoms are co-segregated at central and sub-central layers of Mo GB, the free energy of co-segregation at the central layer is 0.34 eV lower than that at the sub-central layers. It indicates that co-segregation at the central layer is more stable, which we established the GB model based on. The separation work ($W_{sep})$ of the Mo GBs represents the difference in work of fracture between the doped and undoped GBs, which can be defined using the following equation^4^:

$W_{sep}=\frac{E_{FS1}+E_{FS2}-E_{GB}}{S}$ (1)

where $E_{FS1}$ and $E_{FS2}$ are the total energies of two free surfaces generated after the fracture of Mo GBs, respectively, $E_{GB}$ is the total energy of the Mo GB, and $S$ is the area of the fractured GB free surfaces. Herein, a positive separation work $W_{sep}$ reflects that the impurity atoms at the GBs are energetically favorable for making a stronger GB against the intergranular fracture.

As a comparison in Supplementary Fig. 15b, the relative separation work of GB with segregated one interstitial O atoms, regarding the separation work (4.15 eV/Å^2^) of the pure Mo Ʃ3(111) GB, are calculated to be -5.78 %, which is in good agreement with the previous study^5^. It is noticed from Supplementary Fig. 15c that the segregation of four doped Ni and Fe atoms leads to a great increase in the fracture strength of the GB, and the effect of Fe is stronger than Ni, which is accord with previous research^1^. Co-segregation of trace amounts of Ni and O was detected at HAGBs, so the effect of Ni and O co-segregation at the GB is also investigated. As the number of segregated O atoms increases, the relative separation work of GBs can be reduced gradually from -5.78 % to -25.66 %. When one interstitial O atom is co-segregated with doped Ni atoms at the GB, the relative separation work first decreases and then increases to 4.20 % with three doped Ni atoms. For the four substituted Ni atoms-strengthened GB, the presence of interstitial O atoms remarkably reduces the relative separation work of the GB from -1.21 % to -24.3 %, and the effect becomes more pronounced with a higher number of O atoms. Above DFT studies clearly demonstrate that increasing the amount of O at the GB reduces the GB cohesion strength notably^5^, and thus in practice, reducing the O concentration should be a key strategy to enhance the GB fracture strength. Also, it should be noted that Ni substitution can reduce the negative effect of interstitial O on GB strength to some extent, which well explains that the B-1200 sample with trace Ni and ultra-low concentration O element co-segregation has significantly stronger GB cohesion strength.

The impact of segregated atoms on GBs of Mo is further analyzed by the Electron Localization Functional (ELF) calculation, which can reflect the distribution of electron density and bonding characteristics at GBs^5^. Supplementary Fig. 15c plots the 2D ELF plots at GBs for a deeper understanding of electron localization caused by the segregation of O and Ni atoms around Mo atom at the GB. The range of ELF is between 0 and 1; ELF = 0 indicates electron complete delocalization and no bonding between the adjacent atoms, and ELF = 1 suggests that the electrons are more localized between the adjacent atoms^6^. The color around the interstitial O atom is orange (about 0.8) but that between the Mo and Ni atoms is close to wathet (0.3-0.4), representing that the outer electrons of the adjacent metallic atoms are captured by O and a typical ionic bond is formed. Thus, with the segregation of O, a depletion zone of charge density between Mo and O atoms is formed, which causes the weakening of Mo-O bonding strength^5–9^. Nevertheless, the ELF values around Ni atoms between the adjacent host metal atoms are about 0.3, very close to that between the adjacent Mo atoms, which would induce the formation of a new stronger Mo-Ni metallic bonds. The co-segregation of O and Ni at Mo GB shows that the electron localization in the blue region between Mo-O is weakened, due to the additional free electrons brought by the segregated Ni atoms at the GBs, such that the brittleness of the GBs caused by Mo-O bonding is decreased to some extent, contributing to an enhanced intergranular fracture resistance of Mo.

Generally, a higher bond order signifies a stronger chemical bond^10,11^. We employed bond order of the core structure at GB center in Supplementary Fig. 16 and Supplementary Table 1, which quantitatively characterize the bond strength of Mo GB with the segregation of O and Ni. The bond order of Mo-Mo in the Mo bulk and the GB center are 0.43~0.52 and 0.34~0.38, respectively. As depicted in Supplementary Fig. 16b, the bond order of the central Mo atom (No. 1) and the Ni atoms (No. 11, 12, 13) is 0.471, which are remarkably surpassed that of the upper (No. 6, 7, 8) and lower (No. 2, 3, 4) neighboring Mo atoms with band order of 0.350 to 0.389.

The introduction of O atom (No. 13) in both GB+1O_int_ and GB+4Ni_sub_+1O_int_ generates a formidable bond with the central Mo atom with a respective bond order of 0.432 and 0.498 portrayed in Supplementary Fig. 16a and 16c. Meanwhile, a noteworthy shift in the bond strength occurs between the center Mo and the adjacent Mo atoms (No. 4, 8) with the bond order diminished to 0.172/0.203 in GB+1O_int_. Similar change occurred in the GB+4Ni_sub_+1O_int_ case. This indicates that the conclusion of the band order is consistent with the ELF calculation.

**Supplementary References**

1. Scheiber, D., Pippan, R., Puschnig, P., Ruban, A. & Romaner, L. Ab-initio search for cohesion-enhancing solute elements at grain boundaries in molybdenum and tungsten. *Int. J. Refract. Met. Hard Mater.* **60**, 75-81 (2016).

2. Ma, H. *et al.* Segregation of interstitial light elements at grain boundaries in molybdenum. *Mater. Today Comm.* **25**, 101388 (2020).

3. Scheiber, D., Romaner, L., Pippan, R. & Puschnig, P. Impact of solute-solute interactions on grain boundary segregation and cohesion in molybdenum. *Phys. Rev. Mater.* **2**, 093609 (2018).

4. Rice, J. R. & Wang, J.-S. Embrittlement of interfaces by solute segregation. *Mater. Sci. Eng. A* **107**, 23-40 (1989).

5. Jing, K. *et al.* Excellent high-temperature strength and ductility of the ZrC nanoparticles dispersed molybdenum. *Acta Mater.* **227**, 117725 (2022).

6. Wang, Z. *et al.* Solving oxygen embrittlement of refractory high-entropy alloy via grain boundary engineering. *Mater. Today* **54**, 83-89 (2022).

7. Sanyal, S., Waghmare, U. V., Subramanian, P. R. & Gigliotti, M. F. X. First-principles understanding of environmental embrittlement of the Ni/Ni_3_Al interface. *Scr. Mater.* **63**, 391-394 (2010).

8. Sanyal, S., Waghmare, U. V., Hanlon, T. & Hall, E. L. Ni/boride interfaces and environmental embrittlement in Ni-based superalloys: A first-principles study. *Mater. Sci. Eng. A* **530**, 373-377 (2011).

9. Zhao, D., Løvvik, O. M., Marthinsen, K. & Li, Y. Segregation of Mg, Cu and their effects on the strength of Al Σ5 (210)[001] symmetrical tilt grain boundary. *Acta Mater.* **145**, 235-246 (2018).

10. Manz, T. A. Introducing DDEC6 atomic population analysis: part 3. Comprehensive method to compute bond orders. *RSC Adv.* **7**, 45552-45581 (2017).

11. Song, J. *et al.* Thermal instability originating from the interface between organic-inorganic hybrid perovskites and oxide electron transport layers. *Energy Environ. Sci.* **15**, 4836-4849 (2022).
